# An integrated molecular cytogenetic map of *Cucumis sativus *L. chromosome 2

**DOI:** 10.1186/1471-2156-12-18

**Published:** 2011-01-27

**Authors:** Yonghua Han, Zhonghua Zhang, Sanwen Huang, Weiwei Jin

**Affiliations:** 1 National Maize Improvement Center of China, Key Laboratory of Crop Genetic Improvement and Genome of Ministry of Agriculture, Beijing Key Laboratory of Crop Genetic Improvement, China Agricultural University, Beijing 100094, PR China; 2 State Key Laboratory of Plant Cell and Chromosome Engineering, Institute of Genetics and Developmental Biology, Chinese Academy of Sciences, Beijing 100101, PR China; 3 Key Laboratory of Horticultural Crops Genetic Improvement of Ministry of Agriculture, Sino-Dutch Joint Lab of Horticultural Genomics Technology, Institute of Vegetables and Flowers, Chinese Academy of Agricultural Sciences, Beijing 100081, PR China

## Abstract

**Background:**

Integration of molecular, genetic and cytological maps is still a challenge for most plant species. Recent progress in molecular and cytogenetic studies created a basis for developing integrated maps in cucumber (*Cucumis sativus *L.).

**Results:**

In this study, eleven fosmid clones and three plasmids containing 45S rDNA, the centromeric satellite repeat Type III and the pericentriomeric repeat CsRP1 sequences respectively were hybridized to cucumber metaphase chromosomes to assign their cytological location on chromosome 2. Moreover, an integrated molecular cytogenetic map of cucumber chromosomes 2 was constructed by fluorescence *in situ *hybridization (FISH) mapping of 11 fosmid clones together with the cucumber centromere-specific Type III sequence on meiotic pachytene chromosomes. The cytogenetic map was fully integrated with genetic linkage map since each fosmid clone was anchored by a genetically mapped simple sequence repeat marker (SSR). The relationship between the genetic and physical distances along chromosome was analyzed.

**Conclusions:**

Recombination was not evenly distributed along the physical length of chromosome 2. Suppression of recombination was found in centromeric and pericentromeric regions. Our results also indicated that the molecular markers composing the linkage map for chromosome 2 provided excellent coverage of the chromosome.

## Background

Cucumber (*Cucumis sativus *L., 2n = 2x = 14) is an economically important vegetable crop in the Cucurbitaceae family. The cucumber genome has been sequenced using a novel combination of traditional Sanger and next-generation Illumina GA sequencing technologies [1]. Illumina GA sequencing technology has significantly improved high throughput sequencing efforts at reasonable cost. However, an intrinsic characteristic of the technology is short read lengths (~50 bp), which prevents their direct application for *de novo *genomic assembly. Within a total of 72.2-fold genome coverage generated for cucumber genome, Sanger reads provided 3.9-fold coverage and Illumina GA reads provided 68.3-fold coverage [1]. The total length of assembled cucumber genome was 243.5 Mb which is 30% smaller compared to cucumber genome size. Of these, only 72.8% of the assembled sequences were anchored onto the chromosomes using information from high density genetic map previously developed by Ren et al. [2]. However, the genetic map reports only the linear order of markers and the amount of recombination between linked markers. Because linkage map distances are not simply related to physical distances, the linkage map does not provide sufficient detail to support genome assembly. The molecular cytogenetic map incorporating data from both genetic and cytological maps can provide sufficient detail of the physical locations of genetic markers. Such maps can contribute significantly to the assembly of ongoing cucumber genomic sequences by resolving the order of closely linked markers, confirming the physical positions of markers on the linkage groups and evaluating the size of the putative remaining gaps [3,4].

The direct way to generate a cytogenetic map is to localize genetic markers onto chromosomes by fluorescence *in situ *hybridization. However, most genetic markers (0.5-4.0 kb) are too small to generate consistent and reliable *in situ *hybridization signals on plant chromosomes [5]. Large insert DNA clones, such as bacterial artificial chromosome (BAC) or yeast artificial chromosomes (YAC) clones, are likely to contain dispersed repetitive sequences that will cause high background signal in FISH [6]. BACs from species such as wheat, with very large genomes do not generate unique locus-specific FISH signals [7]. Small fosmid clones (30-40 kb) likely to contain less dispersed repetitive sequences compared to large insert DNA clones, will be more suitable as DNA probes. A fosmid library was recently constructed for *C. sativus *inbred line 9930 which was previously used for International Cucumber Genome Project. A high-density polymorphic simple sequence repeat (SSR) genetic map was developed based on whole genome shotgun sequences [2]. In addition, a karyotype showing the position and fluorescence intensity of signals generated by several tandem repeat sequences has been developed for *C. sativus *inbred 9930 [8]. These accomplishments have created the basis for the integration of molecular, genetic and cytological maps of cucumber. FISH mapping of DNA clones anchored with genetically mapped DNA markers to pachytene bivalents is a very efficient approach to integrate genetic linkage maps with chromosomal maps [9]. Not only do the pachytene chromosomes provide superior mapping resolution compared to somatic metaphase chromosomes, but the euchromatin and heterochromatin features can be visualized on pachytene chromosomes, thereby allowing DNA probes to be mapped to specific euchromatic or heterochromatic regions. To date, FISH-based cytogenetic maps on pachytene chromosomes have been developed in *A. thaliana *for chromosome 4 [10], maize chromosome 9 [11,12], potato chromosome 6 [13,14], *Brassica oleracea *chromosome 6 [15], rice chromosomes 5 and 10 [4,16], tomato chromosomes 1, 2 and 6 [17-19], soybean chromosome 19 [20], cotton chromosomes 12A and 12D [21] and for all the *Sorghum *chromosomes [22,23]. We previously reported integrated cytogenetic maps for cucumber chromosomes 6 and 7 [24]. Here, we report an integrated cytogenetic map for cucumber chromosome 2 using similar methods as described previously [24].

## Results

### The distribution of 45S rDNA, Type III and CsRP1 sequences on cucumber metaphase chromosome 2

**Figure 1:**
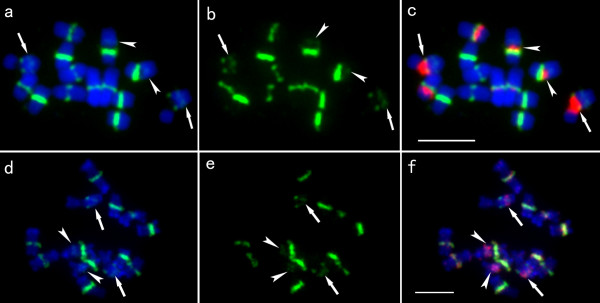
**Distribution of 45S rDNA, Type III and CsRP1 sequences on cucumber metaphase chromosomes 2 and 4**. **a-b **Minor Type III (green) signals in the interstitial regions of chromosome pairs 2 (arrows) and 4 (arrowheads). **c **The 45S rDNA (red) signals occupied the exclusive constriction region and the signals of major and minor Type III satellite repeat flanked the constriction on chromosome pairs 2 (arrows) and 4 (arrowheads). **d-f **The minor Type III (green) signals were colocalized with strong signals of the CsRP1 (red) on chromosome pairs 2 (arrows) and 4 (arrowheads). *Bars*, 5 μm

Our previous study demonstrated that the satellite repeat sequence Type III located at cytologically defined cucumber centromeres, and the Type III signals on chromosome 2 were the weakest among the seven chromosome pairs [8]. In this study, we found that minor Type III signals also occurred at the interstitial regions of chromosome pairs 2 (arrows in Figure 1a, b) and 4 (arrowheads in Figure 1a, b) which were identified by FISH mapping of the 45S rDNA probe simultaneously on optimal chromosome preparations (Figure 1c). The minor signals on chromosome 4 (arrowheads in Figure 1b) were much weaker than those on chromosome 2 (arrows in Figure 1b). Interestingly, an unusual distribution of the 45S rDNA sequence was found. The signals of 45S rDNA, which are usually associated with a secondary constriction, occupied the exclusive constriction region. The major and minor signals of Type III sequence, which located at the primary constriction regions on other cucumber chromosomes, flanked the constriction on chromosome pairs 2 (arrows in Figure 1c) and 4 (arrowheads in Figure 1c). Moreover, the minor Type III signals on the two chromosome pairs colocalized with strong signals of the CsRP1 sequence (Figure 1d-f), which is a pericentromeric heterochromatin specific repeat [25]. The stronger signals of CsRP1 were close the minor Type III signals on chromosome 2 (arrows in Figure 1d-f).

### The construction of molecular cytogenetic map of chromosome 2

To construct a cytogenetic map with a high resolution, an initial set of 16 SSR markers from linkage group 2 [2] was selected to screen a fosmid library developed from the cucumber inbred line 9930. The SSR markers were distributed at an average distance of ~6 cM along the linkage group from 0.0 cM (bin 1) to 100.2 cM (bin 113). Only 11 fosmid clones were selected for FISH mapping which produced little or no background signal when hybridized to cucumber chromosomes without the aid of *C*_0_*t-*1 DNA for blocking (Table 1).

**Table 1:** Genetic and physical locations of SSR markers and their corresponding fosmid clones

Code	Marker	position (cM)	Fosmid clone	**FL**^***a***^
2-1	SSR00184	0.0	gcfbe0_0022_B06.ab1	1.02
2-2	SSR11952	5.9	gcfbd0_1078_H03.ab1	5.78
2-3	SSR21090	11.1	gcfbd0_0606_B07.ab1	8.49
2-4	SSR13504	22.5	gcsxc0_157514	16.99
2-5	SSR22083	33.6	rgcfbe0_0466_E02.ab1	23.85
2-6	SSR03758	57.5	rgcfbe0_0464_B06.ab1	42.99
2-7	SSR23732	61.5	rgcfbd0_0252_E04.ab1	73.85
2-8	SSR20045	74.0	gcfbd0_1142_B06.ab1	80.34
2-9	SSR06678	78.0	rgcfbd0_0512_G07.ab1	84.28
2-10	SSR30665	94.3	gcfbd0_0304_A09.ab1	96.21
2-11	SSR13783	97.7	gcfbd0_0605_C06.ab1	94.21

^*a *^FL (fraction length) = (S/T) × 100.2, where S = distance (μm) from the FISH site to the end of the long arm, T = total length of the chromosome (μm), and 100.2 is the length (in cM) of the linkage group 2

**Figure 2:**
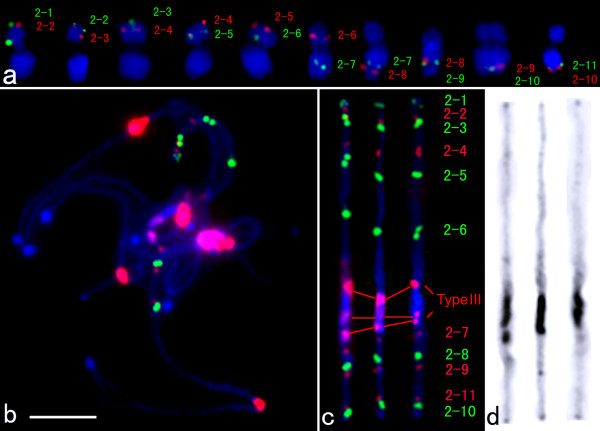
**FISH of 11 fosmids on cucumber somatic and pachytene chromosomes**. **a **FISH of 11 fosmids on somatic chromosomes. Adjacent probes were labeled with different fluorochromes and hybridized together to test the order and position. **b **Cucumber chromosomes at the pachytene stage were probed by a set of eleven fosmid clones together with the Type III satellite repeat. **c **Three straightened cucumber pachytene chromosome 2. The left chromosome is straightened from the image shown in Figure 2b. **d **The chromosomes in Figure 2c were converted into black-white image. Distinct heterochromatin is visible at the centromeric region and at the distal ends of both chromosome arms. *Bars*, 5 μm

We first determined the physical order of adjacent fosmid clones based on the genetic positions of their corresponding SSR markers by dual-color FISH on somatic metaphase chromosomes (Figure 2a). On the basis of these results, multi-fosmid FISH probe cocktails were developed and hybridized to the pachytene chromosomes together with the cucumber centromere-specific DNA probe Type III (Figure 2b). The relative position of all probes can be clearly distinguished on spreads of pachytene bivalents. Three computationally straightened chromosomes 2 from three independent cells were shown (Figure 2c). The left chromosome is straightened from the image shown in Figure 2b. The DAPI-stained pachytene chromosomes in Figure 2c were converted into a black-white image to show heterochromatin distribution (Figure 2d). Pachytene chromosome 2 showed a distinct heterochromatin and euchromatin distribution pattern based on DAPI staining. The heterochromatic region spanned ~18% of the length of the chromosome. Most of the heterochromatin was confined to the pericentromeric region. Small heterochromatic domains were observed at both ends of the chromosome (Figure 2d). Using the FISH signals from Type III repeat as a reference, the centromere for chromosome 2 was placed between the fosmids 2-6 and 2-7.

The order of individual fosmids along the chromosome was generally colinear with the order of the corresponding SSR markers along the linkage map except for 2-10 (94.3 cM) and 2-11 (97.7 cM). The FISH signals derived from fosmid clones 2-10 and 2-11 were located adjacently on pachytene chromosomes but 2-10 was located closer to the telomere (Figure 2c). We examined information from the genome sequence in the region that showed conflict between the genetic map and our FISH result. We found that genome sequence information supported our FISH result (unpublished data). The conflict may be either due to a result of chromosomal rearrangement that occurred between the sequenced genotype 9930 and the genotypes used to create the mapping population or these markers may have been placed incorrectly on the genetic map.

**Figure 3:**
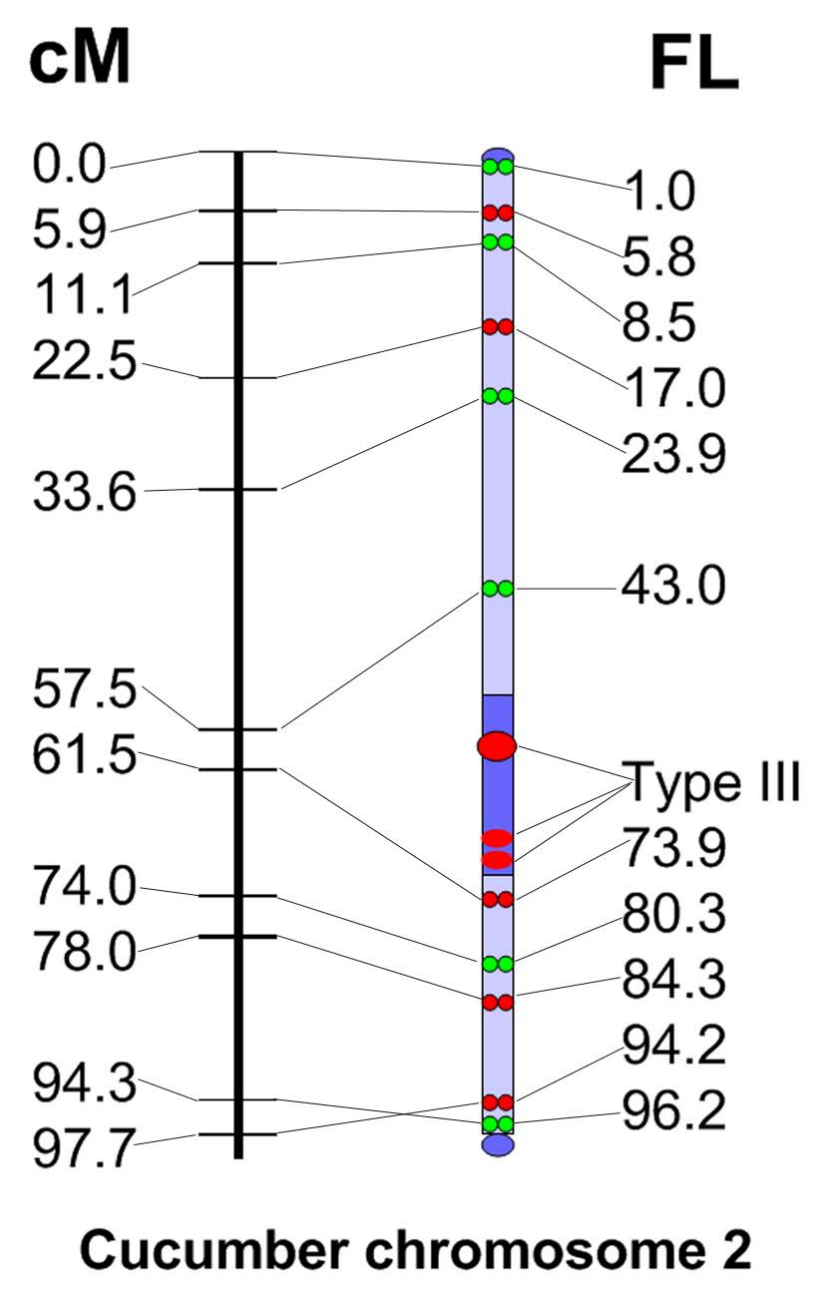
**Comparison of the genetic linkage map and pachytene FISH-based cytogenetic map for cucumber chromosome 2**. The linkage map was drawn according to Ren et al. [2]. The position of each fosmid on the pachytene chromosome is calculated as the fractional length using a methodology reported by Cheng et al. [4]. FL (fraction length) = (S/T) × 100.2, where S = distance (μm) from the FISH site to the end of the long arm, T = total length of the chromosome (μm), and 100.2 is the length (in cM) of the linkage group 2

To assess the rate of recombination, measurements of fosmid positions along the six straightened chromosomes 2 were taken and transformed into fractional lengths (FL) using a methodology reported by Cheng et al. [4]. The relationship between the genetic and physical distances along chromosome 2 is summarized in Table 1 and Figure 3. In general, recombination is more or less evenly distributed along the physical length of chromosome 2 (Figure 3). However, a significant disproportion between genetic and physical distances was found in the centromeric region. SSR03758 (57.5 cM) and SSR23732 (61.5 cM) are the closest SSR markers flanking the centromere. These two markers are separated genetically by only 4 cM but physically by 30.9 FL.

One of the most important criteria to judge the quality of a genetic linkage map is its physical coverage of the corresponding chromosome. The FISH signals for two fosmids (2-1 and 2-10) were very near on the opposing ends of the chromosome. These results indicate that the molecular markers composing the linkage map for chromosome 2 provide excellent coverage of the chromosome.

## Discussion

### Centromere position for cucumber chromosome 2

The centromere is the most characteristic landmark of monocentric chromosomes in higher eukaryotic species and appears cytologically as a distinct primary constriction on condensed metaphase chromosomes. Satellite DNA and retrotransposons are the most abundant DNA elements found in plant centromere regions [26]. Due to large arrays of highly homogenized satellite repeats, elucidating the assembly and structure of chromatin at centromeres is extremely difficult. Thus, the centromeres have been left as gap in the sequence maps in most model eukaryotes. rDNA sequences and several types of satellite sequences primarily located in the centromeric and telomeric regions comprised the majority of unassembled reads in cucumber genome shotgun sequences [1]. FISH is a powerful technique to delineate the structure and DNA composition of such genomic regions [9].

Results of the cytogenetic studies on cucumber chromosome 2 have been inconsistent in previous karyotypes [8,25,27,28], due to different positions assumed for centromere of chromosome 2. In our study, FISH results showed an unusual distribution of the 45S rDNA sequence and the existence of minor centromere-specific satellite loci on chromosome 2, which may have led to confusion about the actual centromere position on this chromosome. The centromere of chromosome 2 was previously assigned to positions which correspond to the chromosomal regions of either the 45S rDNA, the major or the minor Type III repeat signals [8,25,28]. CENH3 is a good marker for assaying centromere activity since it is found only in functional centromeres in eukaryotes [29-35]. The preparation of anti-CENH3 antibody in cucumber would be used to prove the location of functional centromere on cucumber chromosome 2.

### Relationship between genetic and physical distance for cucumber chromosome 2

Our fosmid map of cucumber chromosome 2 revealed a significant disproportion between genetic and physical distances in the centromeric region. Indeed, reduction of recombination around the centromere is a common feature, especially for grasses with large genomes and high repetitive DNA content, such as wheat, barley, and sorghum. Crossing-over in the (peri-)centromeric regions, which may account for as much as 50% of the chromosome length, is essentially suppressed [23,36-40]. In genomes with smaller chromosomes, such as rice and Arabidopsis, cross-over suppression at the centromere is restricted within a relatively small region [4,41,42]. Like rice and Arabidopsis, the region of recombination suppression on chromosome 2 is also restricted to a relatively small region corresponding to ~30% of the chromosome length. Since the region of recombination suppression correlates directly with sizes of centromeric heterochromatin regions [4], the regions of recombination reduction from other cucumber chromosomes should be smaller than the region on chromosome 2 because the proportion of centromeric heterochromatic regions on chromosome 2 is the highest one of all cucumber chromosomes [25]. This has been confirmed by our previous study which demonstrated that an obvious recombination reduction was not detected for cucumber chromosome 6. Also the region of suppressed recombination of chromosome 7 was smaller than that of chromosome 2 [24].

The integrated genetic and cytogenetic maps can serve as a template to facilitate sequence assembly because the maps provided information on the distribution of heterochromatin, euchromatin, centromeres and markers across chromosomes [4,43]. In addition, the cytogenetic maps can be used to determine if the linkage gaps represent recombination hot spots or large chromosomal segments. The assembled cucumber sequences were anchored onto the chromosomes using a high-density genetic map information developed by Ren et al. [2]. Although the genetic linkage map contains more markers per centimorgan than any other cucumber genetic map, it still contains large gaps. Integrated molecular cytogenetic maps can indicate whether these gaps are associated with recombination hot spots and/or represent a large chromosomal segment. Such information will be valuable for designing corresponding strategies to eventually close these gaps.

## Conclusions

Using a combination of molecular, genetic and cytological approaches to the analysis of cucumber genome, we built an integrated map of cucumber chromosome 2, to provide also an explanation for previous different localization of the centromere of this chromosome and find that the meiotic recombination frequency is reduced around the cucumber centromeres. With the establishment of cytogenetic maps of other cucumber chromosomes, this integrated set of information will not only provide a framework for cucumber genome assembly but also provide a solid foundation for cucumber genetic and genomic research such as map-based gene isolation, comparative genomics and evolutionary studies.

## Methods

### Plant materials and chromosome preparation

*C. sativus *'Chinese long' inbred line 9930 was used for cytological studies. Root tips were harvested from germinated seeds, pretreated in 0.002M 8-hydroxyquinoline at room temperature for 2 h to accumulate metaphase cells, and fixed in methanol:glacial acetic acid (3:1). Root tips were macerated in 2% cellulase Onozuka R-10 (Yakult Pharmaceutical, Tokyo) and 1% pectolyase Y-23 (ICN) at 37°C for 2 h and squashes were made in the same fixative. Young panicles were harvested and fixed in 3:1 (100% ethanol:glacial acetic acid) Carnoy's solution. The procedure for meiotic chromosome preparation was largely the same as that used for preparing mitotic chromosomes from root tips with the following modification: anthers were digested in the enzyme mixture for 4.5 h at 37°C. The digested anthers were macerated on glass slides in 50% acetic acid solution with fine-pointed forceps and then flame-dried.

### Fluorescence *in situ *hybridization (FISH)

All fosmid clones were provided by the Beijing Genomics Institute, Beijing, China. The fosmid library was constructed from genomic DNA of inbred line 9930 which was also used for whole genome sequencing. SSR markers spaced ~6 cM apart across linkage groups 2 [2] were used to select fosmids for FISH. Fosmid DNA was isolated using QIAGEN plasmid midi kit and further purified by Plant DNeasy spin columns (QIAGEN). The 45S rDNA, Type III and CsRP1 clones of cucumber [8] were used. FISH was performed according to published protocols [44]. DNA probes were labeled with digoxigenin-dUTP or biotin-dUTP via nick translation and detected with antidigoxigenin antibody coupled with Rhodamine (Roche) or avidin-conjugated with FITC (Vector Laboratories), respectively. Chromosomes were counterstained by 4,6-diamidino-2-phenylindole (DAPI) in a VectaShield antifade solution (Vector Laboratories). Images were captured digitally using a CCD camera (QIMAGING, RETIGA-SRV, FAST 1394) attached to an Olympus BX61 epifluorescence microscope. Gray-scale images were captured for each color channel and then merged. Chromosome straightening was performed using the 'straighten-curved-objects' plug-in of Image J [45], and final image optimization was performed using Adobe Photoshop (Adobe Systems).

## Authors' contributions

YH, SH and WJ designed research. YH and ZZ performed research. YH and WJ analyzed data and wrote the paper. All authors read and approved the final manuscript.
